# Negative target stimuli do not influence cross-modal auditory distraction

**DOI:** 10.1371/journal.pone.0274803

**Published:** 2022-10-07

**Authors:** Saskia Kaiser, Axel Buchner, Laura Mieth, Raoul Bell

**Affiliations:** Department of Experimental Psychology, Heinrich Heine University Düsseldorf, Düsseldorf, Germany; University of Zurich, SWITZERLAND

## Abstract

The present study served to test whether emotion modulates auditory distraction in a serial-order reconstruction task. If auditory distraction results from an attentional trade-off between the targets and distractors, auditory distraction should decrease when attention is focused on targets with high negative arousal. Two experiments (with a total *N* of 284 participants) were conducted to test whether auditory distraction is influenced by target emotion. In Experiment 1 it was examined whether two benchmark effects of auditory distraction—the auditory-deviant effect and the changing-state effect—differ as a function of whether negative high-arousal targets or neutral low-arousal targets are used. Experiment 2 complements Experiment 1 by testing whether target emotion modulates the disruptive effects of reversed sentential speech and steady-state distractor sequences relative to a quiet control condition. Even though the serial order of negative high-arousal targets was better remembered than that of neutral low-arousal targets, demonstrating an emotional facilitation effect on serial-order reconstruction, auditory distraction was not modulated by target emotion. The results provide support of the automatic-capture account according to which auditory distraction, regardless of the specific type of auditory distractor sequence that has to be ignored, is a fundamentally stimulus-driven effect that is rooted in the automatic processing of the to-be-ignored auditory stream and remains unaffected by emotional-motivational factors.

## Introduction

It is well established that auditory distraction impairs immediate memory for visually presented stimuli [1] which is referred to as the irrelevant-sound effect. One of the standard paradigms to investigate this type of cross-modal auditory distraction is the serial-recall paradigm in which participants have to serially recall a sequentially presented list of visual targets (e.g., digits, consonants, or words) that are presented while task-irrelevant auditory distractors have to be ignored [2–4]. In addition to the serial-recall task, other tasks that rely on serial-order processing such as serial-order reconstruction tasks [e.g., 5–7]—in which the targets are re-presented at test and their order has to be reproduced—, yielded results parallel to those obtained in the serial-recall task. Theories of auditory distraction focus on the processing requirements of the primary task [e.g., 8, 9] and on the specific properties of the to-be-ignored information that is thought to be responsible for the distraction [e.g., the degree to which the to-be-ignored information deviates from a previous train of stimuli; see, for example, 10, 11]. As yet, comparatively little attention has been devoted to emotional-motivational factors that could influence cross-modal auditory distraction. When examining auditory distraction, emotions are often seen as extraneous factors that researchers try to remove from the equation by creating emotionally sterile laboratory settings. The task-relevant target material often consists of random permutations of digits and consonants and is thus stripped of emotional content and arousal. Usually, it is seen as sufficient to instruct participants to attend to the visual targets and to ignore the auditory distractors, despite the dullness of the to-be-remembered material. This approach rests on the assumption that participants unquestioningly adopt the notions of relevance and irrelevance conveyed by the instructions. However, in our everyday experience, the relevance or irrelevance of information is not only determined by extrinsic rules and conventions (e.g., the teacher instructing the students to focus on the school subject) but also by the intrinsic properties of the information on which attention is supposed to be focused (e.g., whether the school subject is Latin or pop culture). It is well possible that people may be particularly prone to distraction when the to-be-attended target material is of little intrinsic relevance to them. It thus seems conceivable that target emotion may be a key factor in modulating distraction. Therefore, the aim of the present experiments was to provide an empirical test of whether cross-modal distraction in the serial-order reconstruction paradigm is modulated by the emotional properties of the target materials.

### Effects of target emotion on attention and memory

Before presenting specific hypotheses about the effects of emotion on auditory distraction, it is useful to briefly provide some background knowledge on the effects of target emotion on cognition. Emotional stimuli are intrinsically relevant because they directly relate to the organism’s ultimate goals of survival and reproduction [12–14]. Emotional stimuli are therefore often postulated to be prioritised in attention and memory [13, 15–20]. This processing advantage is usually attributed to emotional and, in particular, negative arousal [16, 17, 21, 22]. For instance, a memory advantage for negatively and positively arousing words over neutral words has been shown in free recall [e.g., 17, 22, 23, 24] and recognition tasks [e.g., 16, 22].

There is some evidence that emotion can affect serial recall, but the evidence is mixed. In two studies [25, 26] a serial-recall advantage for emotional words compared to neutral words was found. In another study no difference in serial recall between positive and negative words was found [27]. An important difference between these studies is that arousal was allowed to differ between the emotional and non-emotional word lists in the study in which an emotional modulation of serial recall was found [26; there is no information on arousal in 25] whereas there was no effect of valence on serial recall when arousal did not differ between positive and negative targets [27]. As yet, there are no studies on the potential influence of emotional targets on auditory distraction in a serial-recall task or a serial-order reconstruction task.

### Theoretical accounts of auditory distraction

The primary aim of the present study was to examine, for the first time, the effect of target emotion on auditory distraction in a serial-order reconstruction task. The question of whether and, if so, how target emotion affects auditory distraction can help to refine theoretical accounts of auditory distraction. Specifically, the effect of cross-modal distraction is often ascribed to an attentional trade-off between the deployment of attention to the primary task and the allocation of attention to the task-irrelevant modality [28–30]. This *attentional-trade-off view* implies that auditory distraction should decrease when attention is closely focused on the primary task. Thus, if the to-be-attended material intrinsically attracts attention, then the focus of attention should be less likely to be diverted by auditory distractors. The attentional-trade-off view thus predicts that auditory distraction should be decreased for negative high-arousal targets. Negative arousal should bind attention to the primary task of remembering the targets, which should, in turn, decrease the chances of auditory distractors receiving attention.

An alternative view is that attention is captured by the distractors in a primarily stimulus-driven manner [31–33]. According to the *automatic-capture account*, the automatic detection of changes and unexpected events in the auditory modality consumes processing resources regardless of the level of engagement in the primary task. As a consequence, distraction should occur independently of the emotional-motivational significance of the targets. The automatic-capture account thus predicts that auditory distraction should be unaffected by target emotion.

A third possibility is that the effect of emotion depends on the type of auditory distractor that has to be ignored. Specifically, one of the most popular theories of auditory distraction, the *duplex-mechanism account* [34–36], is based on the core assumption that there are two types of auditory distraction. First, *interference-by-process* may be the automatic consequence of the involuntary processing of the auditory distractors which cannot be controlled and does not depend on the level of engagement afforded by the primary task. Second, *attentional capture* may result from an attentional trade-off between the deployment of attention to the primary task and the allocation of attention to the task-irrelevant modality. *Specific* attentional capture occurs when the content of the sound is inherently salient such as one’s own name or the sound of one’s own child [34, 37]. *Aspecific* attentional capture occurs when the content of the sound is not inherently salient but gains salience based on the context in which it occurs such as a deviant sound in a series of repetitions of one stimulus [34]. Both types of attentional capture are assumed to be amenable to cognitive control.

According to the duplex-mechanism account, the *changing-state effect* [38, 39] is a prime example of interference-by-process. It is well established that changing-state sequences consisting of different distractor stimuli (e.g., F G C D E A B H) disrupt performance more than steady-state sequences consisting of a repeated distractor stimulus (e.g., A A A A A A A A). The duplex-mechanism account postulates that, when changes between consecutive distractors in the acoustic stream are registered, the order of these changes is automatically processed. These changes are postulated to be present only in changing-state sequences but not in steady-state sequences. The automatic processing of the order of the changing distractors interferes with the voluntary processing of the order of the target sequences. The obligatory nature of the underlying processes should guard the effect against emotional-motivational influences. In contrast, the *auditory-deviant effect*, defined as the more disruptive effect of auditory-deviant sequences compared to steady-state sequences [40, 41], is attributed to aspecific attentional capture. In auditory-deviant sequences, one of the distractors deviates from the rest of the distractor sequence (e.g., A A A A A B A A). According to the duplex-mechanism account, the violation of an expectation about the continuation of the sequence triggers attentional engagement which draws attention away from the primary task. Importantly, the account entails the assumption that attentional diversion by auditory deviants is under cognitive control which provides a basis for the influence of emotional-motivational factors. Specifically, the account implies that attentional diversion “can be influenced by a range of factors including task demands, emotional state, and motivational factors” [34, p. 33].

### Emotional-motivational influences on auditory distraction

The empirical support for these theoretical accounts with regards to emotional-motivational factors is mixed. Recently, Bell et al. [32] have reported that providing external monetary incentives for good performance improved serial recall but influenced neither the size of the changing-state effect nor the size of the auditory-deviant effect, suggesting that the influence of top-down motivational factors on auditory distraction is limited. By contrast, other studies have reported that visually masking the target stimuli decreases the disruptive effects of auditory deviants and emotional distractors [35, 42]. These findings were explained by assuming that the perceived difficulty of encoding visually masked stimuli triggers an upregulation of task engagement which decreases attentional diversion by shifting the attentional resources to the target stimuli. However, these findings have been called into question by Kattner and Bryce [43] who consistently failed to replicate the suppressive effect of visual masking on the auditory-deviant effect across four experiments.

Even fewer data are available regarding the possible influence of emotional factors on auditory distraction. Most research was focused on emotional properties of the auditory distractors [42, 44–48] with the dominant finding being that distractors with emotional characteristics capture more attention than emotionally neutral distractors. The question of whether emotional characteristics of the primary task affect cross-modal distraction is much less well researched. Recently, Kaiser et al. [33] have demonstrated, in a series of four well-powered experiments, that positive and negative mood states affect neither the changing-state effect nor the auditory-deviant effect, thereby providing evidence in support of the automatic-capture account [32] according to which cross-modal attention is rooted in the automatic detection of auditory changes and deviations and thus independent of emotional states. In that study, however, the emotional states were manipulated by standard mood-induction procedures that were applied either before the serial-recall task or in-between the serial-recall trials. Selective attention may thus have remained unaffected by the mood manipulation for the simple reason that the emotional arousal of the mood induction was not directly related to the to-be-recalled targets. In fact, whereas the negative or positive mood states were clearly detectable in the participants’ mood ratings, there was no conclusive evidence that the negative or positive mood states were reflected in the serial-recall performance at all. With no such effect in the performance measures, it is possible to conclude that negative and positive mood had no apparent effect on the degree to which participants were engaged in the primary task.

Different results may occur when the targets themselves are emotional. Specifically, attention may be attracted more strongly to targets with emotional significance which may increase memory for the targets and decrease cross-modal distraction. There is, in fact, evidence suggesting that psychophysiological correlates of attention switching are affected when emotional targets are used, but the pattern of results is inconsistent across studies. Research focusing on the P3 component of the event-related potential in response to distractor sounds—which is often thought to be associated with attentional orienting to task-irrelevant sounds [49]—has demonstrated a reduced P3 [50, 51] as well as an increased P3 [52–54] in response to novel sounds when the participants’ attention was focused on negative or positive in comparison to neutral visual targets. Neither valence nor the specific type of emotion involved in these studies seem to discriminate reliably between studies showing reduced or increased P3 responses to the emotional stimuli. Thus, the available evidence is not yet conclusive with respect to the question of whether and, if so, how emotional characteristics of the target stimuli affect auditory distraction.

### The present study

Here, we test the effect of target emotion on auditory distraction in the serial-order reconstruction paradigm. To increase the chances of finding significant effects of target emotion on serial-order reconstruction, we aimed at constructing word lists with strong differences in both valence and arousal. Given that negative words in the database that we used [55] were associated with higher arousal than positive words, we decided to contrast negative word lists with high arousal to neutral word lists with low arousal. We expected serial-order reconstruction to be improved for the negative high-arousal word lists [25, 26] but the main and novel question of the present study was whether and, if so, how cross-modal auditory distraction would be affected by the emotional prioritization of the processing of the targets. This question is relevant for theoretical models of auditory distraction as the aforementioned models make diverging predictions about the influence of emotional factors, as explained below.

Experiment 1 is focused on the changing-state effect and the auditory-deviant effect which have both acquired the status of benchmark findings in research on working memory [56]. Participants were asked to ignore steady-state, auditory-deviant, and changing-state sequences. The attentional-trade-off view leads to the prediction that negative high-arousal targets lead to an improved attentional focus on the primary task which implies that distraction in general and thus both the changing-state effect and the auditory-deviant effect should be decreased relative to the control condition with neutral low-arousal targets. The automatic-capture account, by contrast, leads to the prediction that both the changing-state effect and the auditory-deviant effect are unaffected by emotional-motivational factors. Finally, the duplex-mechanism account implies that the auditory-deviant effect should be decreased by the negative arousal of the targets while the changing-state effect should remain unaffected by target emotion, resulting in an interaction between target emotion and distractor type.

When examining auditory-deviant and changing-state effects, it is necessary to use the steady-state condition as a control condition against which the auditory-deviant and changing-state conditions are compared. However, it has been demonstrated that steady-state sequences cause reliable distraction relative to quiet [57]. Therefore, Experiment 2 was designed to compare changing-state and steady-state conditions against a quiet control condition to test whether the irrelevant-sound effect—yet another benchmark finding of research on working memory [56]—and the steady-state effect are modulated by the negative arousal of the targets. This seemed necessary in order to arrive at a more complete understanding of how auditory distraction is modulated by the emotional content of the target stimuli.

## Experiment 1

### Methods

#### Ethics statement

The experiment was approved by the ethics committee of the Faculty of Mathematics and Natural Sciences at Heinrich Heine University Düsseldorf. The research has been performed in accordance with the Declaration of Helsinki and its later amendments. All participants gave written informed consent before participating in the experiment.

#### Participants

A total of 118 participants (90 women), recruited on campus at Heinrich Heine University Düsseldorf prior to the COVID-19 pandemic, took part in the experiment. Their age ranged from 18 to 36 years with a mean of 22 (*SD* = 4) years. All participants were fluent German speakers (107 native speakers) who reported normal or corrected-to-normal hearing and vision. Participants received course credit or a small monetary reward in exchange for participation. To maximize the sensitivity of the analyses, we aimed at maximizing the number of participants during the two weeks that the lab was available. A sensitivity power analysis, performed with G*Power [58], showed that with *N* = 118 and given α = .05, it was possible to detect a target emotion by distractor condition interaction of the size of η_p_^2^ = .12 with a statistical power of 1 – β = .95.

#### Materials

In the serial-order reconstruction task, each target list consisted of either negative high-arousal words or neutral low-arousal words. In each trial, seven words were sampled, without replacement, either from a set of 100 neutral words or from a set of 100 negative words. Only one-syllable and two-syllable German nouns were used. The words were chosen from the Leipzig Affective Norms for German (LANG) database [55]. The word sets were matched for concreteness, word frequency, number of letters as well as number of syllables and, hence, did not differ on any of these variables (all *p*’s > .573, Table 1). The neutral words were of neutral valence (*M* = 5.02, *SD* = 0.17) on a scale ranging from 1 (negative) to 9 (positive) and low arousal (*M* = 2.34, *SD* = 0.23) on a scale ranging from 1 (low arousal) to 9 (high arousal). We deliberately chose a strong manipulation of target emotion by contrasting negative high-arousal material to neutral low-arousal material to maximize the probability to detect an effect of target emotion on auditory distraction. Therefore, the neutral words were compared to words with negative valence (*M* = 3.69, *SD* = 0.42) and high arousal (*M* = 5.12, *SD* = 1.06). The valence of the negative words was significantly lower than the valence of the neutral words, *F*(1, 198) = 873.03, *p* < .001, η_p_^2^ = .82, while arousal was higher for the negative words than for the neutral words, *F*(1, 198) = 662.99, *p* < .001, η_p_^2^ = .77. The word sets are available in the Open Science Framework repository at https://osf.io/z4afx/.

**Table 1:** Means and standard deviations of the controlled dimensions of the word sets taken from the LANG database [55].

Dimension	Neutral Word Set		Negative Word Set	
	*M*	*SD*	*M*	*SD*
Concreteness	4.18	1.62	4.33	2.06
Word Frequency	12.47	1.93	12.54	2.24
Number of Letters	5.93	1.24	5.86	1.19
Number of syllables	1.80	0.40	1.79	0.41

The mean values for concreteness, word frequency, number of letters and syllables were calculated based on the norms provided by the LANG Database [55]. Concreteness was assessed on a scale ranging from 1 (concrete) to 9 (abstract). Word frequency was taken from the *Wortschatz Lexikon* of the University of Leipzig (https://wortschatz.uni-leipzig.de/de).

The auditory-distractor sequences consisted of a set of 12 letters spoken by a female voice and recorded with a 44.1 sampling rate using 16-bit format. The letters were normalized to minimize amplitude differences among the stimuli and played at about 65 dB(A) L_eq_. The letter set consisted of the following monosyllabic consonants: D, F, H, J, M, P, Q, R, S, V, X, Z. For steady-state sequences one letter was randomly drawn from the set of letters and repeated 12 times. Auditory-deviant sequences were identical to steady-state sequences with the following exception: Randomly determined, the letter at the sixth, seventh or eighth position in the distractor sequence was replaced by a different letter from the set of letters, functioning as the auditory deviant. For changing-state sequences, the 12 letters were presented as distractors in random order.

#### Procedure

**Fig 1:**
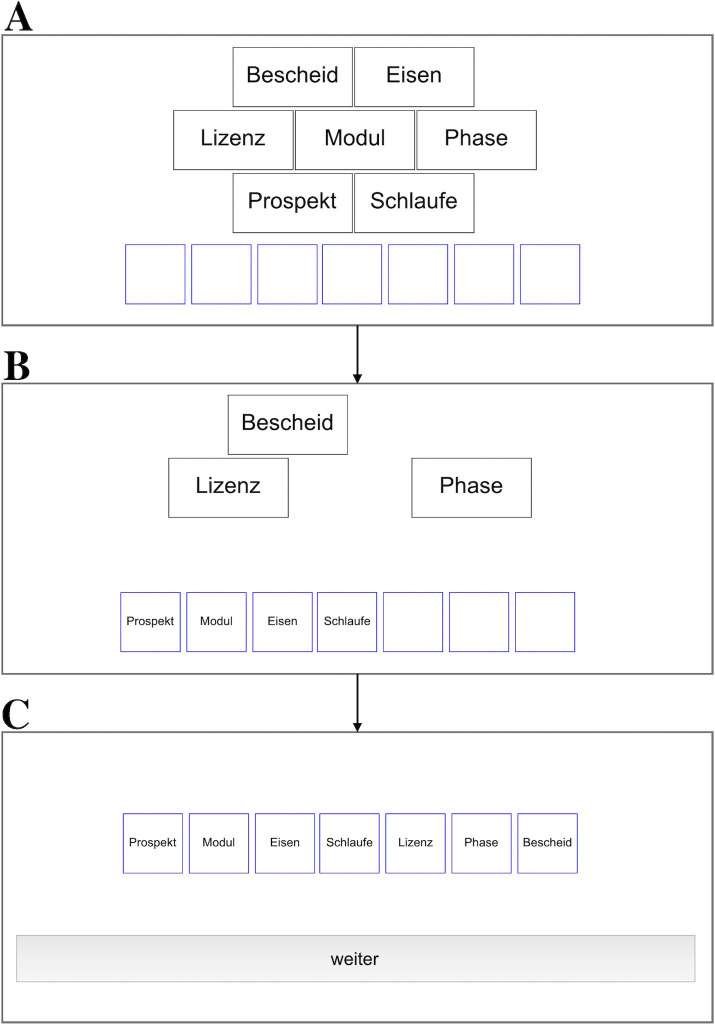
Illustration of the serial-order reconstruction task. A neutral target sequence serves as example: *Bescheid* [notice], *Eisen* [iron], *Lizenz* [license], *Modul* [module], *Phase* [phase], *Prospekt* [brochure], *Schlaufe* [loop]. (A) Immediately after the target sequence had been presented, the target words were presented in alphabetical order in black frames, along with seven blue-framed answer boxes representing the serial positions. (B) By clicking on the alphabetically ordered words, the words appeared successively in the answer boxes representing the serial positions. (C) When all target words were assigned to serial positions, the *weiter* [continue] button was presented, allowing the participants to initiate the next trial.

Throughout the entire experiment, participants wore headphones with high-insulation hearing protection covers (beyerdynamic DT-150) that were directly plugged into the Apple iMac computer running the experiment. At the beginning of the experiment, participants received standardized written instructions on the computer screen. They were instructed to focus on the words displayed on the screen and to ignore the auditory input that would be played via the headphones. They were assured that the auditory input was completely irrelevant for the task and would remain task-irrelevant throughout the whole experiment. Participants then performed 16 serial-order reconstruction training trials to familiarize themselves with the task. The training trials consisted of steady-state trials, half of which consisted of neutral target words and half of negative target words. The data of these trials were not analyzed. The experiment proper consisted of 48 trials. The trials were presented in a random order. There were six target-distractor combinations: neutral targets and steady-state sequences, neutral targets and auditory-deviant sequences, neutral targets and changing-state sequences, negative targets and steady-state sequences, negative targets and auditory-deviant sequences, negative targets and changing-state sequences. There were eight trials of each target-distractor combination, given that a previous study [31] has shown that the ratio of changing-state or deviant trials to steady-state trials has no influence on auditory distraction. In each trial, seven target words were presented, one after another, in black 80 pt Monaco font against a white background at the center of the computer screen. Each of the seven target words was presented for one second with an inter-target interval of 500 ms. The presentation of the distractor sequence started with the onset of the first target word. The auditory distractor letters were presented at a rate of one letter every 875 ms, resulting in a total duration of 10.5 s per distractor sequence. The duration of the target sequence was thus identical to that of the distractor sequence. Immediately after the presentation of the target sequence, all words of the target sequence were displayed on screen in alphabetical order, along with seven empty answer boxes representing the serial positions of the targets (Fig 1). The participants’ task was to reconstruct the order of the target sequence by clicking on the target words in the correct order. As soon as the participant clicked on a target word, the target word disappeared from the list of alphabetically ordered words and appeared in the leftmost empty answer box. It was not possible to skip a position or correct a response. If participants did not know which of the words had been presented at a particular serial position, they had to guess. When all target words had been assigned to a serial position, participants initiated the next trial by clicking a continue button. The whole experiment lasted about 25 minutes on average. The software running the experiment was written in LiveCode (Version 9, available at https://livecode.com).

### Results

The data were analyzed using the MANOVA approach to repeated-measures analyses [59]. All multivariate test criteria correspond to the same exact *F* statistic which is reported. Partial eta squared (η_p_^2^) is reported as a sample effect size measure. All analyses were carried out using IBM SPSS Statistics 28. The dataset of the experiment is available in the supplementary online material in the Open Science Framework repository at https://osf.io/z4afx/.

**Fig 2:**
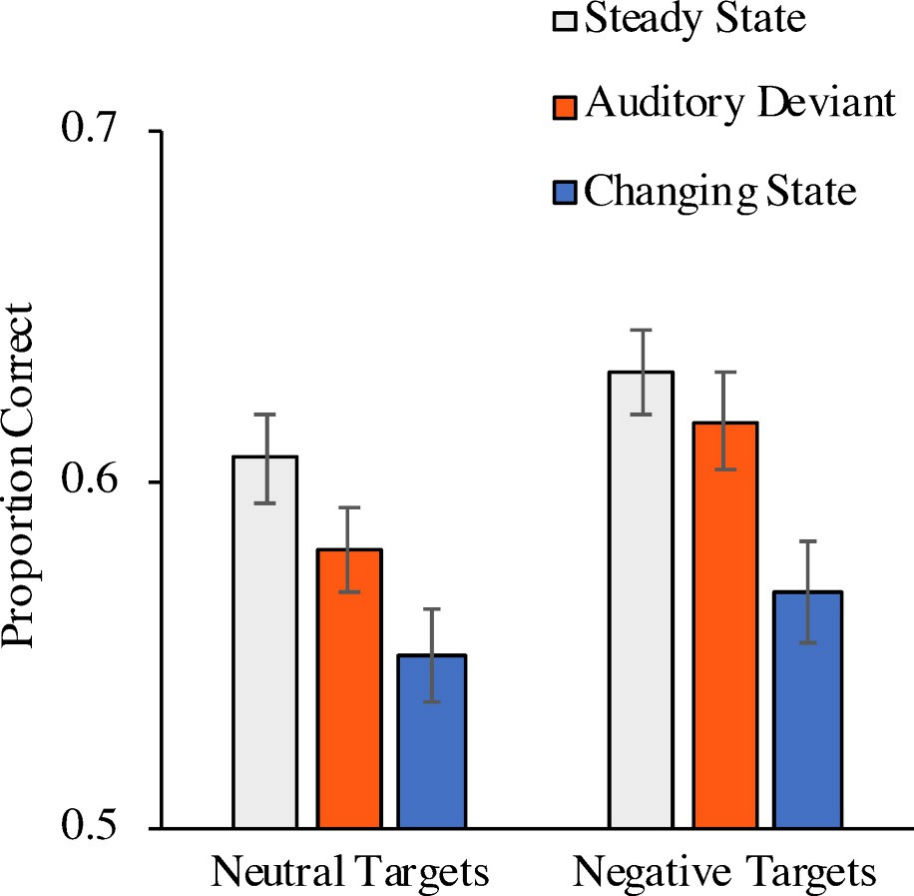
Proportion of correct responses as a function of distractor condition and target emotion (Experiment 1). The error bars represent the standard errors of the means.

A 2 × 3 repeated-measures analysis with target emotion (neutral, negative) and distractor condition (steady state, auditory deviant, changing state) as repeated-measures variables and the proportion of words placed at the correct serial position as dependent variable showed significant main effects of target emotion, *F*(1, 117) = 14.01, *p* < .001, η_p_^2^ = .11, in that the serial order of the negative targets was significantly better remembered than the serial order of the neutral targets, and of distractor condition, *F*(2, 116) = 26.44, *p* < .001, η_p_^2^ = .31 (Fig 2). The interaction between target emotion and distractor condition was not significant, *F*(2, 116) = 0.66, *p* = .521, η_p_^2^ = .01.

Two further analyses were conducted to separately analyze the auditory-deviant effect and the changing-state effect. When the steady-state condition was contrasted with the auditory-deviant condition, there was evidence of an auditory-deviant effect, *F*(1, 117) = 6.83, *p* = .010, η_p_^2^ = .06. Serial-order reconstruction was enhanced for negative targets in comparison to neutral targets, *F*(1, 117) = 12.02, *p* < .001, η_p_^2^ = .09, but the size of the auditory-deviant effect did not differ as a function of target emotion, *F*(1, 117) = 0.54, *p* = .464, η_p_^2^ < .01. When the steady-state condition was contrasted with the changing-state condition, there was evidence of a changing-state effect, *F*(1, 117) = 51.82, *p* < .001, η_p_^2^ = .31. Serial-order reconstruction was enhanced for negative targets in comparison to neutral targets, *F*(1, 117) = 6.68, *p* = .011, η_p_^2^ = .05, but the size of the changing-state effect did not differ as a function of target emotion, *F*(1, 117) = 0.18, *p* = .669, η_p_^2^ < .01.

In addition, two supplementary Bayesian analyses were conducted, using the default settings of JASP [60, 61], to separately test whether the sizes of the auditory-deviant effect and the changing-state effect differed between trials with neutral and negative targets. For this purpose, the auditory-deviant effect and the changing-state effect were computed by subtracting the performance in each condition from the performance in the steady-state condition. Then, two non-directional Bayesian *t*-tests were run to compare the size of the auditory-deviant effect as well as the changing-state effect between the two target emotions. For the auditory-deviant effect, the resulting Bayes factor *BF*_01_ = 7.52 provides moderate evidence in favor of the null-hypothesis. For the changing-state effect, the resulting Bayes factor *BF*_01_ = 8.95 also provides moderate evidence in favor of the null-hypothesis [61].

### Discussion

Overall, target emotion had a positive effect on serial-order reconstruction. Given that there was no interaction between distractor condition and target emotion, the hypothesis of a differential emotional modulation of the auditory-deviant effect and the changing-state effect has to be rejected. Both the auditory-deviant effect [40, 41] and the changing-state effect [38, 39] were reliably obtained. However, none of these effects was significantly modulated by target emotion.

A potential limitation of Experiment 1 is that there was no quiet control condition. When examining changing-state and auditory-deviant effects, the steady-state condition serves as the standard reference condition [56]. It has long been assumed that steady-state distractors cause “little if any disruption compared with quiet” [e.g., 34, p. 31]. This earlier conclusion has been falsified by recent evidence showing that steady-state sequences produce robust disruption of serial recall in comparison to a quiet control condition [57]. However, if steady-state sequences disrupt performance relative to a quiet control condition, logic dictates that the changing-state effect (that is, the difference between the changing-state condition and the steady-state condition) necessarily reflects only a part of the disruption caused by changing-state distractors relative to quiet. This implies that the examination of the modulation of auditory distraction by target emotion provided by Experiment 1 is incomplete. It remains to be tested whether target emotion may modulate the disruption of performance by auditory distractors relative to a quiet condition.

We used two types of distractors in Experiment 2, steady-state and changing-state sequences. Steady-state sequences consisted of identical monosyllabic words. Changing-state sequences consisted of sentential speech. Sentential speech typically causes particularly large amounts of disruption in serial-order memory [e.g., 62] which is most likely not due to semantic or syntactic processing given that reversed speech has been found to cause about the same amount of distraction as forward speech [63, 64, but see 65]. Instead, it is very likely that the greater acoustical complexity of the sentential speech (that is, the greater number of changes in frequency, amplitude, and timing) is primarily responsible for the increase in distraction. Nevertheless, we used reversed speech and reversed monosyllabic words to avoid having to rely on external evidence for the argument that semantic or syntactic distractor properties cannot play a role in the disruption of serial-order memory in the present Experiment 2.

## Experiment 2

### Methods

#### Ethics statement

The experiment was approved by the ethics committee of the Faculty of Mathematics and Natural Sciences at Heinrich Heine University Düsseldorf. The research has been performed in accordance with the Declaration of Helsinki and its later amendments. All participants gave written informed consent before participating in the experiment.

#### Participants

A total of 167 participants took part in the experiment. One dataset had to be excluded prior to analysis because the person had participated twice (the dataset of the first participation was retained). The final sample consisted of 166 participants (118 women) who were recruited on campus at Heinrich Heine University Düsseldorf prior to the COVID-19 pandemic. All participants reported normal or corrected-to-normal hearing and vision and were fluent German speakers (152 native speakers). Their age ranged from 18 to 35 years with a mean age of 23 (*SD* = 4) years. We aimed to recruit at least as many participants as in Experiment 1 and continued data collection until the end of the week in which this goal was reached. A sensitivity power analysis showed that given *N* = 166 and α = .05, it was possible to detect a target emotion by distractor condition interaction of the size of η_p_^2^ = .09 with a statistical power of 1 – β = .95.

#### Materials and procedure

Materials and procedure were identical to those used in Experiment 1 with the following exceptions. The disruptive effects of reversed-speech and steady-state distractor sequences were compared to a quiet control condition, resulting in the following six target-distractor combinations: neutral targets and quiet, neutral targets and steady-state sequences, neutral targets and reversed speech, negative targets and quiet, negative targets and steady-state sequences, negative targets and reversed speech. In the quiet condition, no distractors were played during the presentation of the target sequence. The distractor sequences that were used in the reversed-speech and steady-state conditions were identical to those that had been used in previous studies [63, 66]. The 12 reversed-speech sequences consisted of reversed German sentences spoken by a male voice and recorded with a 44.1 sampling rate using 16-bit format. Correspondingly, there were 12 steady-state sequences. For each steady-state sequence, one monosyllabic word was taken from one of the changing-state sequences and repeated 18 times (corresponding to the mean number of words in the sentences). The steady-state and reversed-speech sequences lasted eight seconds.

As in Experiment 1, each target word was presented for one second, but the inter-target interval was decreased to 150 ms to match the duration of the target sequences to the duration of the distractor sequences.

### Results

**Fig 3:**
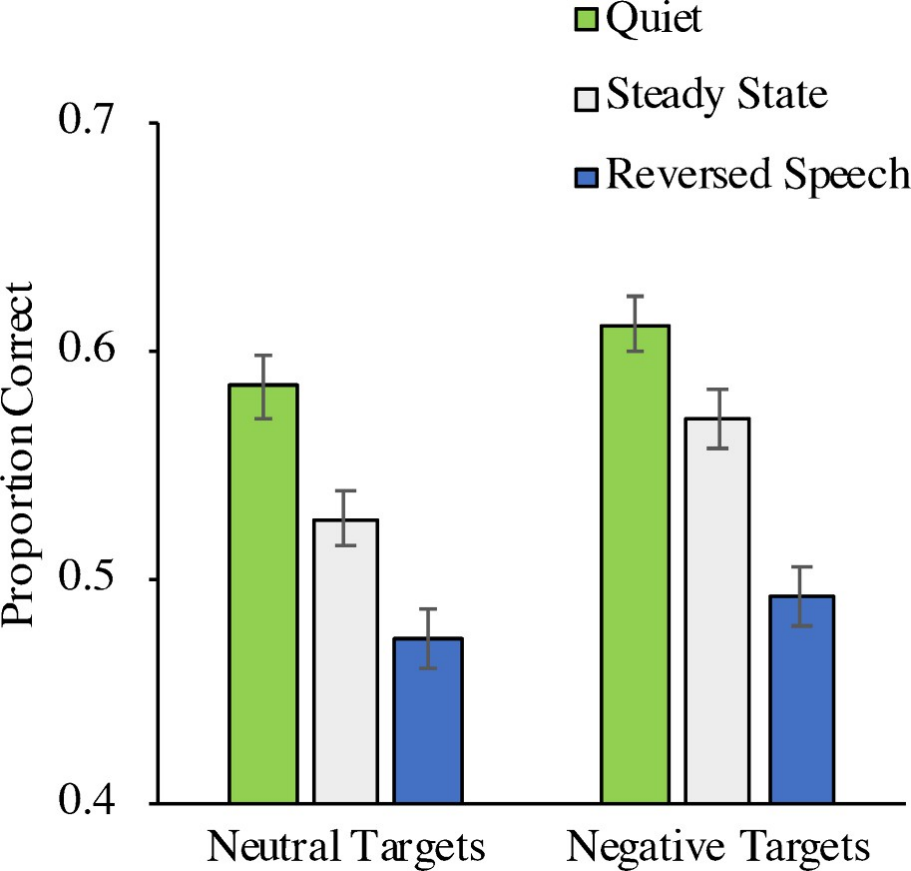
Proportion of correct responses as a function of distractor condition and target emotion (Experiment 2). The error bars represent the standard errors of the means.

A 2 × 3 repeated-measures analysis with target emotion (neutral, negative) and distractor condition (quiet, steady state, reversed speech) as repeated-measures variables and the proportion of words placed at the correct serial position as the dependent variable showed a significant main effect of target emotion, *F*(1, 165) = 26.17, *p* < .001, η_p_^2^ = .14, in that the serial order of the negative targets was significantly better remembered than the serial order of the neutral targets, and of distractor condition, *F*(2, 164) = 90.67, *p* < .001, η_p_^2^ = .53 (Fig 3). The interaction between target emotion and distractor condition was not significant, *F*(2, 164) = 1.70, *p* = .186, η_p_^2^ = .02.

Two further analyses were performed to separately analyze the steady-state effect and the irrelevant-sound effect. When the quiet condition was contrasted with the steady-state condition, there was evidence of a steady-state effect, *F*(1, 165) = 53.38, *p* < .001, η_p_^2^ = .24. Serial-order reconstruction was enhanced for negative targets in comparison to neutral targets, *F*(1, 165) = 22.33, *p* < .001, η_p_^2^ = .12, but the size of the steady-state effect did not differ as a function of target emotion, *F*(1, 165) = 1.84, *p* = .177, η_p_^2^ = .01. When the quiet condition was contrasted with the reversed-speech condition, there was evidence of an irrelevant-sound effect, *F*(1, 165) = 182.19, *p* < .001, η_p_^2^ = .52. Serial-order reconstruction was enhanced for negative targets in comparison to neutral targets, *F*(1, 165) = 10.42, *p* = .002, η_p_^2^ = .06, but the size of the irrelevant-sound effect did not differ as a function of target emotion, *F*(1, 165) = 0.31, *p* = .581, η_p_^2^ < .01.

Two supplementary Bayesian analyses were conducted to separately test whether the sizes of the steady-state effect and the irrelevant-sound effect differed between trials with neutral targets and trials with negative targets. For this purpose, the steady-state effect and the irrelevant-sound effect were computed by subtracting the performance in each condition (steady state and reversed speech) from the performance in the quiet condition. Then, two non-directional Bayesian *t*-tests were run to compare the size of the steady-state effect as well as the irrelevant-sound effect between the two target emotions. For the steady-state effect, the resulting Bayes factor *BF*_01_ = 4.70 provides moderate evidence in favor of the null-hypothesis. For the irrelevant-sound effect, the resulting Bayes factor *BF*_01_ = 9.94 also provides moderate evidence in favor of the null-hypothesis [61].

### Discussion

As in Experiment 1, serial-order reconstruction was better when the serial order of negative targets had to be remembered than when the serial order of neutral targets had to be remembered. However, both the steady-state effect and the irrelevant-sound effect were unaffected by target emotion. This provides further evidence against the attentional-trade-off view [28–30] and in favor of the automatic-capture account [31–33].

## General discussion

The main purpose of the present study was to test whether and, if so, how emotion affects auditory distraction in the serial-order reconstruction paradigm. However, before discussing the results pertaining to the main research question, it seems important to point out that target emotion had a significant main effect on performance in the serial-order reconstruction task. This is consistent with many findings in the literature suggesting that emotional targets are privileged in attention and memory. For instance, a recall advantage for emotional stimuli in comparison to neutral ones has often been demonstrated [16, 17, 21]. There are only a few studies in which the influence of target emotion on serial-order memory was investigated [25–27]. The results of the two experiments reported here add to the results of these studies by demonstrating that the serial order of negative high-arousal targets is better remembered than that of neutral low-arousal targets. This serial-order reconstruction advantage for negative high-arousal targets is consistent with previous evidence from the serial-recall paradigm suggesting that serial recall is enhanced for emotional high-arousal words in comparison to neutral words [26]. This result contrasts with that of a recent study on the effect of mood on auditory distraction [33] in which it was found that negative arousal per se had little influence on the retention of serial order, when emotional arousal was induced via standard mood-induction procedures that required participants to remember and write down negative life events or concentrate on emotionally arousing scenes. The present experiments differ from those previous experiments in terms of how emotional information processing was induced. The mood states induced by Kaiser et al. [33] were general and not directly associated with the serial-recall task. In the present study, by contrast, the target stimuli were negatively arousing. Together, these results suggest that negative arousal may improve the retention of serial order but only if the negative arousal is directly linked to the to-be-attended targets. Thus, the results of the present study demonstrate that the manipulation of emotional arousal had significant effects on cognitive performance which represents favorable conditions for testing whether auditory distraction is affected by emotion.

### The effect of target emotion on auditory distraction

The main purpose of the present experiments was to test whether target emotion is a determinant of cross-modal auditory distraction in the serial-order reconstruction paradigm. Examining whether or not emotional factors modulate auditory distraction seems interesting from an applied point of view because real-life situations are often emotionally charged but studies on the intrinsic emotional aspects of the to-be-remembered events on cross-modal distraction are lacking. Further, this question is relevant for theoretical models of auditory distraction. These models make diverging predictions about the influence of emotional factors. First, the attentional-trade-off view [28–30] implies that cross-modal distraction is decreased once attention is bound by the emotional arousal of the target stimuli. This prediction is contradicted by the data of both Experiments 1 and 2 in which the effect of auditory distraction was unaffected by target emotion in every condition tested despite the robust modulation of serial-order reconstruction performance by the negative arousal of the targets. The attentional-trade-off view thus fails to account for the present data and its attractiveness as an explanation of cross-modal distraction is weakened. Second, within the automatic-capture account [31–33], it is assumed that cross-modal auditory distraction arises automatically from the obligatory perceptual processing of changes and deviations in the to-be-ignored auditory channel. This account thus implies that cross-modal distraction remains unaffected by the emotional arousal of the target stimuli which is what was observed in all conditions of the present Experiments 1 and 2. It thus has to be concluded that the automatic-capture account is compatible with the results obtained here and its attractiveness as an explanation of cross-modal distraction is strengthened, the more so given that the conceptualization of auditory distraction as a primarily stimulus-driven process has long been prevalent in related paradigms of cross-modal auditory distraction such as the oddball paradigm [10, 11]. Third, the duplex-mechanism account [34] implies that the auditory-deviant effect should be modulated by target emotion whereas the changing-state effect should not be modulated by target emotion. In Experiment 1 the auditory-deviant effect [40, 41] as well as the changing-state effect [38, 39] were replicated. However, none of these effects was modulated by target emotion. This finding is incompatible with the prediction of the duplex-mechanism account [34]. Interestingly, the present results are completely in line with evidence provided by Kattner and Bryce [43]. They have shown that the auditory-deviant effect remains just as unaffected by manipulations of target prioritization as the changing-state effect. The attractiveness of the duplex-mechanism account as an explanation of cross-modal distraction is thus weakened [see also 31–33].

In contrast to what has been stated previously [e.g., 4, 34], evidence from experiments with large sample sizes providing sufficient statistical power suggests that steady-state sequences cause considerable disruption relative to a quiet condition [57]. This was also confirmed by the results of the present Experiment 2 and—in line with the findings of Bell et al. [57]—the sample effect size of the steady-state effect was in fact quite substantial. The present results thus also add to those previous results showing that steady-state distractors cause reliable disruption of serial-order memory.

### Limitations and prospects for future research

The present results necessarily have to be interpreted within the limitations of the specific methods of research used in the present study. For instance, the present experiments were focused on the changing-state effect, the steady-state effect and the auditory-deviant effect—more specifically, the variant of the auditory-deviant effect in which the deviant is defined in terms of acoustic changes relative to the surrounding stimuli [34]. Auditory distraction based on semantic properties of the distractor sequences such as the recently discovered categorial-deviation effect—the variant of the auditory-deviant effect in which the deviant is based on a change of semantic category such as a digit in a sequence of letters [67, 68]—was not included and should be considered in future studies in order to arrive at an even more comprehensive understanding of auditory distraction and its susceptibility to emotional-motivational factors. Furthermore, the present experiments involved only the comparison between neutral low-arousal targets and negative high-arousal targets but they did not include positive emotions. Our study thus cannot provide a complete picture on the effects of emotion on auditory distraction. Given the sparse and partly contradictory evidence regarding the influence of emotional-motivational factors in the serial-recall task [32, 33, 35, 42, 43] further systematic examinations of emotional-motivational factors such as valence, dominance, and performance-related emotional states (e.g., fatigue, boredom, or stress) are desirable to reach more robust conclusions about the underlying mechanisms of auditory distraction.

## Conclusion

In conclusion, negative targets are associated with enhanced immediate memory as reflected in serial-order reconstruction performance compared to neutral stimuli. Nevertheless, task-irrelevant auditory distractors impair serial-order reconstruction performance independently of whether the to-be-remembered targets have a high intrinsic emotional significance or not. This is evidence in favor of the automatic-capture account and against both the attentional-trade-off view and the duplex-mechanism account of auditory distraction. From an applied point of view the pervasive disruption suggests that auditory distraction is a challenge for staying focused even in emotionally engaging situations. Hence, independent of the intrinsic emotional properties of the main task, it is important to protect performance from distracting auditory influences.
